# MiR-422a promotes adipogenesis via MeCP2 downregulation in human bone marrow mesenchymal stem cells

**DOI:** 10.1007/s00018-023-04719-6

**Published:** 2023-02-27

**Authors:** Angelica Giuliani, Jacopo Sabbatinelli, Stefano Amatori, Laura Graciotti, Andrea Silvestrini, Giulia Matacchione, Deborah Ramini, Emanuela Mensà, Francesco Prattichizzo, Lucia Babini, Domenico Mattiucci, Elena Marinelli Busilacchi, Maria Giulia Bacalini, Emma Espinosa, Fabrizia Lattanzio, Antonio Domenico Procopio, Fabiola Olivieri, Antonella Poloni, Mirco Fanelli, Maria Rita Rippo

**Affiliations:** 1 Department of Clinical and Molecular Sciences, Università Politecnica delle Marche, Via Tronto 10/A, Ancona, Italy; 2 SOD Medicina di Laboratorio, Azienda Ospedaliero Universitaria delle Marche, Ancona, Italy; 3 Department of Biomolecular Sciences, Molecular Pathology Laboratory “PaoLa”, University of Urbino Carlo Bo, Fano, PU Italy; 4 Department of Biomedical Sciences and Public Health, Università Politecnica delle Marche, Ancona, Italy; 5 Clinic of Laboratory and Precision Medicine, IRCCS INRCA, Ancona, Italy; 6 IRCCS MultiMedica, Milan, Italy; 7 Section of Hematology, Department of Clinical and Molecular Sciences, Università Politecnica delle Marche, Ancona, Italy; 8 IRCCS Istituto delle Scienze Neurologiche di Bologna, Laboratorio Brain Aging, Bologna, Italy; 9 Geriatrics, Santa Croce Hospital, Azienda Ospedaliera Ospedali Riuniti Marche Nord, Fano, Italy; 10 Scientific Direction, IRCCS INRCA, Ancona, Italy

**Keywords:** Methyl CpG binding protein 2, Mesenchymal stromal cells, Adipogenesis, Osteogenesis, MicroRNA, Osteoporosis

## Abstract

Methyl-CpG binding protein 2 (MeCP2) is a ubiquitous transcriptional regulator. The study of this protein has been mainly focused on the central nervous system because alterations of its expression are associated with neurological disorders such as Rett syndrome. However, young patients with Rett syndrome also suffer from osteoporosis, suggesting a role of MeCP2 in the differentiation of human bone marrow mesenchymal stromal cells (hBMSCs), the precursors of osteoblasts and adipocytes. Here, we report an in vitro downregulation of MeCP2 in hBMSCs undergoing adipogenic differentiation (AD) and in adipocytes of human and rat bone marrow tissue samples. This modulation does not depend on MeCP2 DNA methylation nor on mRNA levels but on differentially expressed miRNAs during AD. MiRNA profiling revealed that miR-422a and miR-483-5p are upregulated in hBMSC-derived adipocytes compared to their precursors. MiR-483-5p, but not miR-422a, is also up-regulated in hBMSC-derived osteoblasts, suggesting a specific role of the latter in the adipogenic process. Experimental modulation of intracellular levels of miR-422a and miR-483-5p affected MeCP2 expression through direct interaction with its 3′ UTR elements, and the adipogenic process. Accordingly, the knockdown of MeCP2 in hBMSCs through MeCP2-targeting shRNA lentiviral vectors increased the levels of adipogenesis-related genes. Finally, since adipocytes released a higher amount of miR-422a in culture medium compared to hBMSCs we analyzed the levels of circulating miR-422a in patients with osteoporosis—a condition characterized by increased marrow adiposity—demonstrating that its levels are negatively correlated with *T*- and *Z*-scores. Overall, our findings suggest that miR-422a has a role in hBMSC adipogenesis by downregulating MeCP2 and its circulating levels are associated with bone mass loss in primary osteoporosis.

**Supplementary Information:**

The online version contains supplementary material available at 10.1007/s00018-023-04719-6.

## Introduction

The methyl-CpG binding protein 2 (MeCP2) is known principally for its ability to inhibit the transcription complex assembly on DNA by binding methylated CpG islands across the genome [1]. Beyond its role in transcriptional repression, more recent studies revealed that MeCP2 may play a complex multifunctional role, coordinating also transcriptional activation, chromatin architecture, and RNA splicing, depending on the molecular context [2–4]. MeCP2 expression is ubiquitous throughout the body, although it is particularly abundant and studied in brain cells. Indeed, in females X-linked mutations of the *MECP2* gene cause Rett syndrome (RTT), a neurodevelopmental disorder characterized by loss of acquired motor and language skills, autistic features, and unusual stereotyped movements [5, 6]. However, the variety of phenotypes identified in RTT patients and MeCP2 mutant mouse models points to important roles for MeCP2 in peripheral systems, including altered lipid metabolism, unbalanced adipose tissue endocrine activity [7, 8], and decreased bone mineral density, among others [9, 10].

In bone marrow (BM), mesenchymal stromal cells (BMSCs), the precursors of adipocytes and osteoblasts, are exposed to a plethora of stimuli that determine the balance between adipogenesis and osteogenesis which in turn are competing and reciprocal [11, 12]. The differentiation of MSCs, in fact, is a two-step process: lineage commitment (from BMSCs to lineage-specific progenitors) and maturation (from progenitors to specific cell types). Studying the mechanisms that regulate bone marrow adipogenesis is important because the marrow adipose tissue (MAT) is not only a passive space-filler. Indeed, MAT actively participates in a broad spectrum of physiological functions—e.g*.* energy homeostasis, immunity, hematopoiesis, coagulation, and regulation of blood pressure—through the release of several molecular mediators [13], including adiponectin, of which BM adipocytes are among the major contributors [14]. Variations in BM adipocyte mass have been reported in primary osteoporosis and other systemic conditions like aging, type 2 diabetes, obesity, myelodysplastic syndrome, cancer therapy, and anorexia nervosa, suggesting that an abnormal differentiation of BMSCs could contribute to pathogenic skeletal manifestations associated with such diseases [15].

Adipogenesis is a finely tuned multi-step process requiring the sequential activation of numerous transcription factors driving the typical physiological and morphological changes observed in the progenitor cells, i.e. cell cycle arrest, metabolic reprogramming, and lipid accumulation [16]. The expression of peroxisome proliferator-activated receptor gamma (PPARγ) is critical to promote fat cell differentiation, and survival of adult adipocytes, inducing the expression of genes involved in insulin sensitivity, lipogenesis, and lipolysis [17–19].

Among the small non-coding RNAs, microRNAs (miRNAs, miRs) represent an additional mechanism for controlling adipogenic gene expression [20]. Given their unique ability to simultaneously regulate multiple protein targets and processes, it has been suggested that miRNAs may play a leading role in BMSC differentiation. Their involvement in adipogenesis has been investigated through experimental and bioinformatic, target-based approaches [21–23]; some identified miRNAs would appear to be involved in lineage commitment while others in maturation. The latter, in some cases, would involve switches that suppress the osteogenic process by activating the adipogenic one [24, 25]. Interestingly, several studies in the nervous system, cancer, and smooth muscle cell differentiation suggest that miRNAs can directly regulate the expression of MeCP2 [26–32].

The role of MeCP2 in the BMSC differentiation process is still unknown but given the relevance of fine control of the transcriptional and epigenetic processes in BMSCs differentiation, it is conceivable that MeCP2 and miRNAs regulating its expression could affect their fate.

Therefore, the aim of our study was to identify miRNAs able to modulate the adipogenic process and their role in the MeCP2-mediated modulation of adipogenesis. For this purpose, we (i) evaluated MeCP2 expression in hBMSCs undergoing differentiation; (ii) screened for miRNAs differentially regulated during hBMSC differentiation in vitro; (ii) validated miRNAs selectively upregulated in adipocytes compared to their precursors; (iii) tested their role in enhancing adipogenesis, and (iv) assessed their ability to modulate MeCP2 expression in hBMSCs. Finally, the levels of circulating miRNAs involved in hBMSC differentiation have been assessed in a cohort of elderly subjects with primary type II osteoporosis, to investigate their association with bone mass loss due to the expansion of the MAT compartment.

## Results

### MeCP2 is downregulated in adipose tissue and during adipogenesis of hBMSC in vitro

**Fig. 1:**
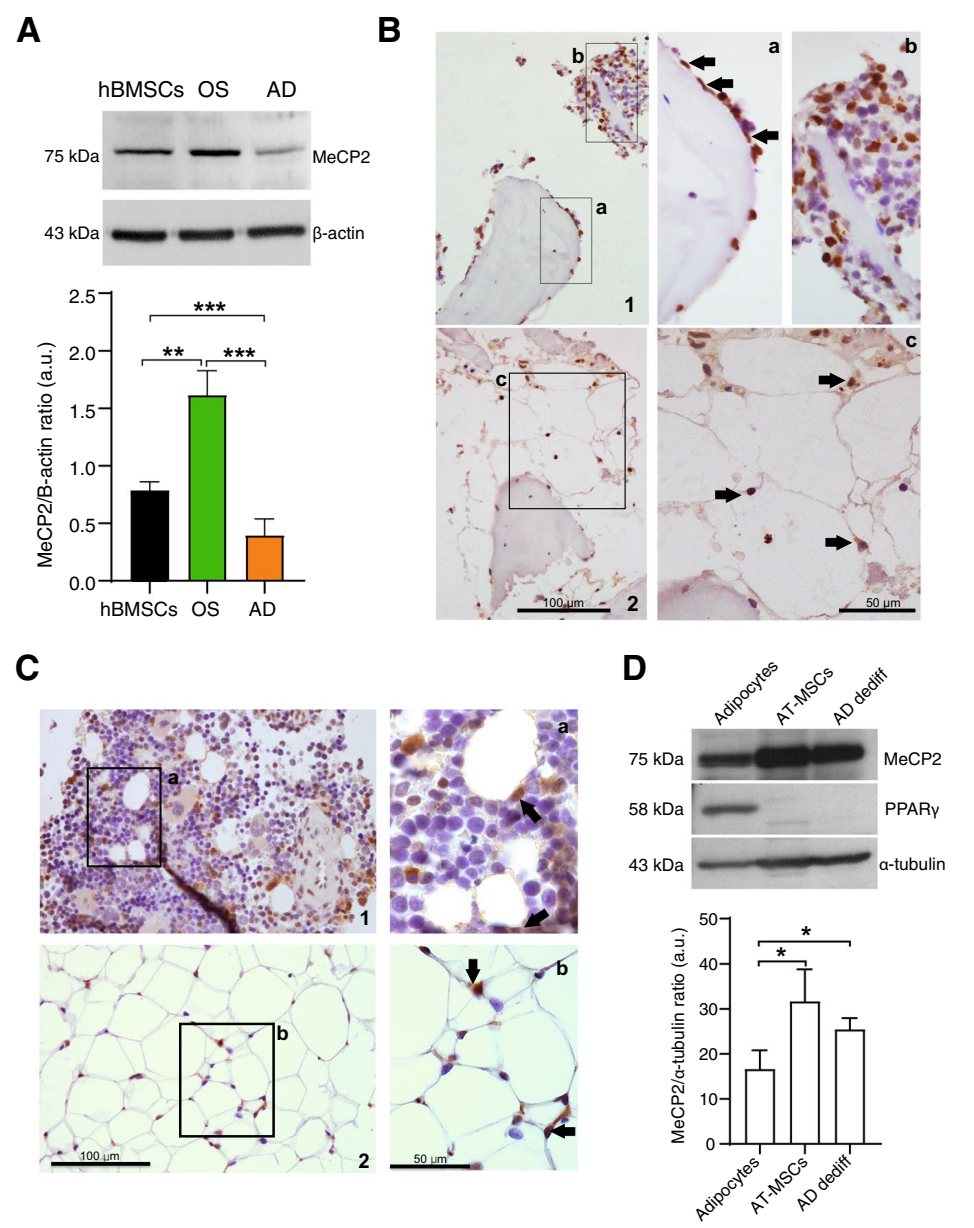
MeCP2 expression in adipocytes and adipogenic process. **A** Western blot and densitometric analysis of MeCP2 in human bone marrow mesenchymal stromal cells (hBMSCs) and hBMSC-derived adipocytes (AD) and osteoblasts (OS) after 14 days of pro-differentiating treatment. Data were normalized to β-actin. **B** Immunohistochemical detection of MeCP2 reactivity in the human femoral bone marrow. 1, Numerous MeCP2-positive nuclei are present. a, Periosteal positive cells (arrows). b, Hematopoietic positive cells. 2, Negative human BM adipose tissue; c, positive hematopoietic cells (arrows) in close contact with adipocyte membrane. Images were taken at 200× and 400× magnification. **C** Immunohistochemical detection of MeCP2 reactivity in rat. 1, Femoral bone marrow, MeCP2 positive hematopoietic cells in close contact with adipocyte membrane are present (a, arrows), no staining was observed in adipocyte nuclei. 2, Inguinal white adipose tissue (AT), low reactivity in adipocytes was confirmed, whereas interstitial and blood cells were positive (b, arrows). Images were taken at 200× and 400× magnification. **D** Western blot and densitometric analysis of MeCP2 and PPARγ expression in human adipocytes, mesenchymal stromal cells (AT-MSCs) and dedifferentiated adipocytes (AD dediff) obtained from subcutaneous adipose tissue of the same donor. Western blot image is relative to 1 out of 3 different analyzed donors. Data were normalized to α-Tubulin. Data are mean ± SD of three independent experiments. *t-test *p* < 0.05; **t-test *p* < 0.01; ***t-test *p* < 0.001

To determine the role of MeCP2 during adipogenesis, MeCP2 expression was analyzed in human bone marrow MSCs induced to differentiate into adipocytes and osteoblast. As shown in Fig. 1A MeCP2 protein expression was downregulated in BMSC-derived adipocytes compared to undifferentiated cells, whereas an opposite modulation was observed in osteoblasts. Furthermore, immunohistochemical (IHC) staining performed in human bone marrow sections obtained from healthy donors revealed a high expression of MeCP2 in the nuclei of periosteal cells (Fig. 1B(a)) and of several hematopoietic cells (Fig. 1B(b)); on the contrary, MeCP2 expression was not found in adipocytes (Fig. 1B(c)). The same results are confirmed in the bone marrow and adipose tissue (AT) samples derived from rats (Fig. 1C). To strengthen this observation, MeCP2 expression was analyzed in human adipocytes and MSCs, isolated from subcutaneous fat of the same donors, in addition to dedifferentiated adipocytes obtained as previously described [33, 34]. Here, we show that MeCP2 expression is significantly lower in adipocytes compared to MSCs and dedifferentiated cells. PPARγ was used as a control of the differentiation state (Fig. 1D).

### MeCP2 downregulation promotes adipogenic program in hBMSCs

**Fig. 2:**
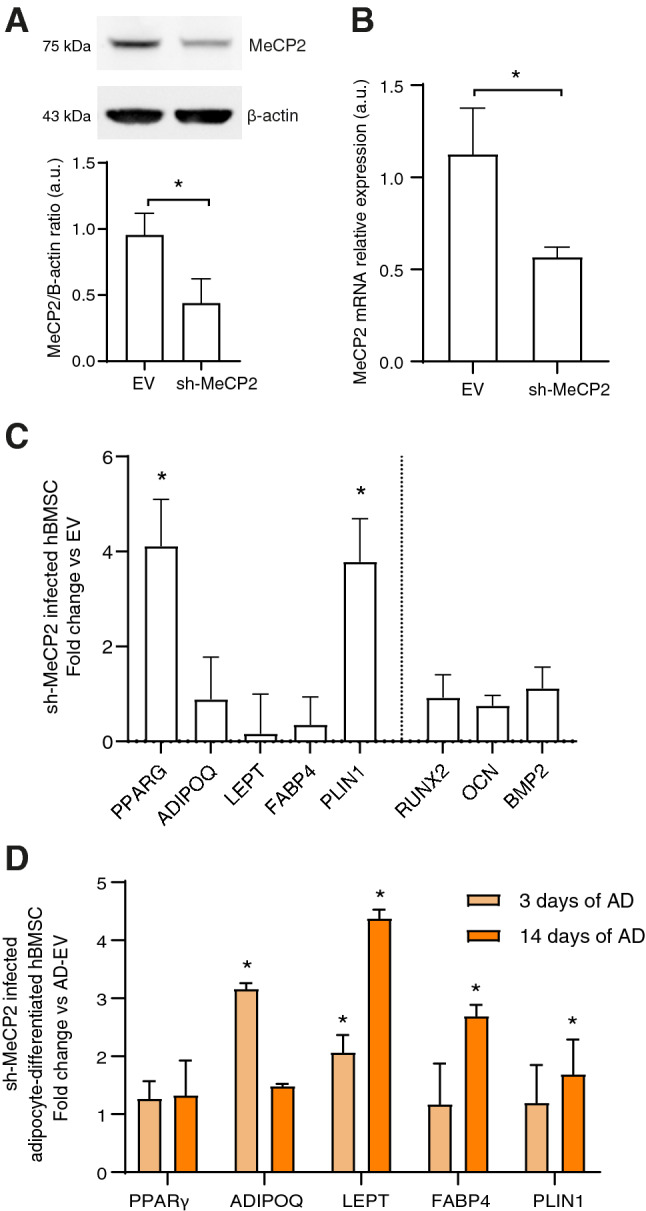
MeCP2 partial silencing induces adipogenesis. MeCP2 expression in undifferentiated hBMSCs infected with shRNA-containing (sh-MeCP2) or empty lentiviral vectors (EV) was analyzed by **A** western blot and densitometric analysis, (data were normalized to β-actin) and **B** by RT-PCR. **C** Adipogenesis (left)- and osteogenesis (right)-related mRNA fold change in hBMSCs infected with shRNA-containing lentiviral vectors *vs* cells infected with empty vector (EV). **D** Adipogenesis-related mRNA fold change in hBMSCs induced to differentiate for 3 and 14 days with shRNA-containing vectors vs hBMSCs induced to differentiate for 3 and 14 days with empty vector (AD-EV). Data are mean ± SD of three independent experiments. *t-test *p* < 0.05

To unravel the role of MeCP2 modulation in adipogenesis, we reduced its expression in hBMSCs by infecting these cells with a pool of three MeCP2-targeting shRNAs (shMeCP2) or control empty-lentiviral vectors (EV). MeCP2 expression was significantly decreased by about 50% both at protein and mRNA levels in undifferentiated shMeCP2-BMSCs compared to EV-BMSCs (Fig. 2A, B). MeCP2 silencing, although showing a not complete abrogation of the protein level, was sufficient to significantly increase PPARγ and PLIN1 mRNA expression in undifferentiated shMeCP2-BMSCs compared to EV-BMSCs (Fig. 2C) and to up-regulate the late adipogenic markers adiponectin and leptin, tested at different time points, once induced to differentiate into adipocytes using complete adipogenic medium (Fig. 2D). Notably, no significant modulation of the osteogenesis-related markers RUNX2, OCN, and BMP2 was observed in shMeCP2-BMSCs (Fig. 2C). Figure 2D shows mRNA levels of genes related to adipogenesis in shMeCP2-BMSCs undergoing adipogenic differentiation. Notably, mRNA of ADIPOQ and LEPT were upregulated after 3 days while LEPT, FABP4, and PLIN1 after 14 days of differentiation in shMeCP2-BMSCs compared to hBMSCs induced to differentiate into adipocytes with empty vector (AD-EV). No significant difference between shMeCP2-BMSCs and AD-EV was observed for PPARγ mRNA expression (Fig. 2D).

### MiR-422a and miR-483-5p are upregulated during adipogenesis and affect MeCP2 expression in hBMSCs and hBMSC-derived adipocytes

**Fig. 3:**
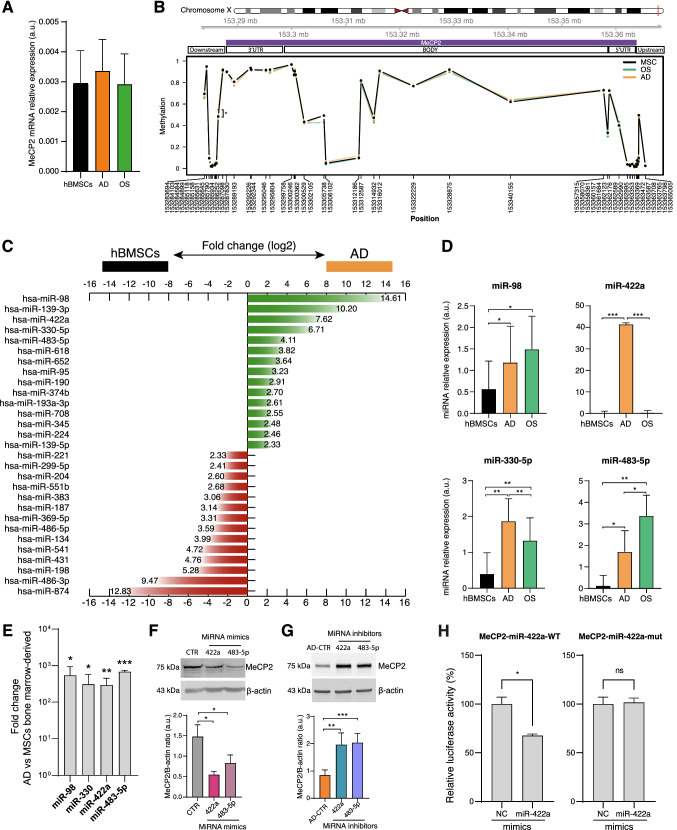
miRNA expression in adipogenesis. **A** MeCP2 mRNA relative expression in arbitrary units (a.u.). Data were normalized to IPO8. **B** DNA methylation profile of the genomic region encompassing MeCP2 (chrX:153282264–153368188, hg19 assembly). The position of microarray probes along the chromosome is reported in the *x*-axis, while the *y*-axis reports DNA methylation levels expressed as beta values, ranging from 0 to 1. **C** Bar chart reporting the (log_2_) fold changes of the miRNAs showing a differential expression in adipocytes (AD) compared to hBMSCs. The figure showed miRNAs with absolute (foldchange) ≥ 5. In green and in red are shown miRNA upregulated and downregulated in adipocytes, respectively. **D** miRNA relative expression validated through Real-time PCR. **E** Fold-change relative expression of miRNA of adipocytes compared with MSCs from the same donors. **F** Western blot and densitometric analysis of MeCP2 in hBMSCs transfected with miRVANA miRNA mimic negative control #1 (CTR) and in hBMSCs transfected with miR-422a and miR-483-5p miRNA mimics. Data were normalized to β-actin. **G** Western blot and densitometric analysis of MeCP2 in adipocytes transfected with miRNA inhibitor negative control #1 (AD-CTR) and in adipocytes transfected with miR-422a and miR-483-5p inhibitors. Data were normalized to β-actin. **H** Luciferase reporter assay. HEK293 cells were infected with either negative control (NC) or miR-422a mimic, then transfected with the luciferase constructs of the wild-type MeCP2 3′-UTR (MeCP2-miR-422a-WT) or a mutated MeCP2 3′-UTR (MeCP2-miR-422a-mut). The luciferase activity was analyzed. *t-test *p* < 0.05; **t-test *p* < 0.01; ***t-test *p* < 0.001

The silencing of MeCP2 demonstrates its role in adipogenesis, therefore, we deeply explored the mechanisms underlying MeCP2 protein expression during hBMSC differentiation. We analyze the MeCP2 mRNA levels and the methylation status of CpGs among a genomic region covering the MeCP2 gene in undifferentiated-BMSCs and cells cultured for 14 days in pro-adipogenic or -osteogenic media. The results showed that MeCP2 modulation in differentiated hBMSCs does not depend on the transcription nor on the methylation status of its gene (Fig. 3A, B).

These observations prompted the hypothesis that the decline in MeCP2 expression during adipogenesis could be related to post-transcriptional mechanisms, i.e. the interaction between miRNAs and MeCP2 mRNA that may inhibit the protein translation. To identify a set of miRNAs potentially involved in BMSC adipogenesis, the differential miRNA expression profiles of hBMSC-derived adipocytes (obtained cultivating hBMSCs in adipogenic medium (AD) for 14 days) and undifferentiated hBMSCs were analyzed by performing a Taqman miRNA qRT-PCR-array. PPARγ and ADIPOQ RT-PCR analysis and fat-soluble staining Oil Red images were used to verify hBMSC differentiation (Supplementary Fig. 1A). Out of 384 tested miRNAs, 15 miRNAs were significantly upregulated (fold change ≥ 5.0), while 14 were downregulated (fold change ≤ −5.0) in AD compared to hBMSCs (Fig. 3C). Complete profiling results have been deposited in NCBI’s Gene Expression Omnibus (GEO) (https://www.ncbi.nlm.nih.gov/geo) with accession reference GSE189508.

In the attempt to identify miRNAs involved in adipogenesis with a possible role in the post-transcriptional modulation of MeCP2, we first proceeded with qPCR validation of the five most upregulated miRNAs (fold change (log_2_) > 4), miR-98, -139-3p, -422a, -330-5p, and -483-5p. The analysis confirmed the results of the microarray with miR-422a showing the highest expression among all miRNAs tested (Fig. 3D). However, miR-139-3p validation yielded high Ct values (> 30) in all conditions tested, suggesting its negligible expression (data not shown).

To identify miRNAs endowed with a pro-differentiating effect and those specific for adipogenesis, we also assessed their expression in osteoblasts obtained by culturing hBMSCs for 14 days in an osteogenic medium (OS) (Fig. 3D). All miRNAs were significantly up-regulated in OS, except for miR-422a which did not show a significant modulation in OS compared to hBMSCs. Osteogenesis was assessed by mRNA analysis of Osteocalcin, BMP2 and Runx2, and Alizarin Red S staining (Supplementary Fig. 1B).

To further confirm these results, the expression of the four miRNAs was tested in human BMSCs and AD isolated from the bone marrow of the same donors. Consistently, the expression of all validated miRNAs was significantly upregulated in isolated AD compared to BMSCs (Fig. 3E).

Overall, these results suggest that miR-98, miR-422a, miR-330-5p and miR-483-5p may have a role in hBMSC differentiation, with miR-422a being more specific for adipogenesis.

To test whether miR-98, miR-422a, miR-330-5p, and miR-483-5p may affect hBMSC differentiation by interacting with MeCP2, we performed a search into the ENCORI miRNA–mRNA interaction database, which collects information from five different prediction tools, observing that MeCP2 mRNA is a predicted target of miR-422a (PITA score = −16.08, interaction supported by 2 Ago CLIP-seq experiments) and miR-483-5p (PITA score = −19.01, Pan-cancer score = 6, interaction supported by 2 Ago CLIP-seq experiments), which has been previously demonstrated to downregulate MeCP2 expression by binding the 3′ UTR of its mRNA [32]. Given the upregulation of the former in differentiated hBMSCs and the specific expression of the latter in hBMSC-differentiated into adipocytes, we focused the subsequent experiments on the role of miR-422a and miR-483-5p.

To investigate whether these two miRNAs are responsible for adipocyte MeCP2 down-regulation, hBMSCs and hBMSC-differentiated adipocytes were transfected for 72 h with specific miRNA mimics or inhibitors, respectively. Figure 3F shows that both miRNA mimics strongly reduced MeCP2 expression in undifferentiated cells. In line with these results, miR-422a and miR-483-5p inhibitors induced upregulation of MeCP2 protein in adipocytes (Fig. 3G). On the other hand, miR-422a and miR-483-5p expression were not significantly modulated by MeCP2 silencing (Supplementary Fig. 2). We also performed a luciferase reporter assay, which confirmed that miR-422a binds at least one of the two 3′ untranslated regions of MeCP2 (Fig. 3H).

### MiR-422a and miR-483-5p promote adipogenesis in hBMSCs

**Fig. 4:**
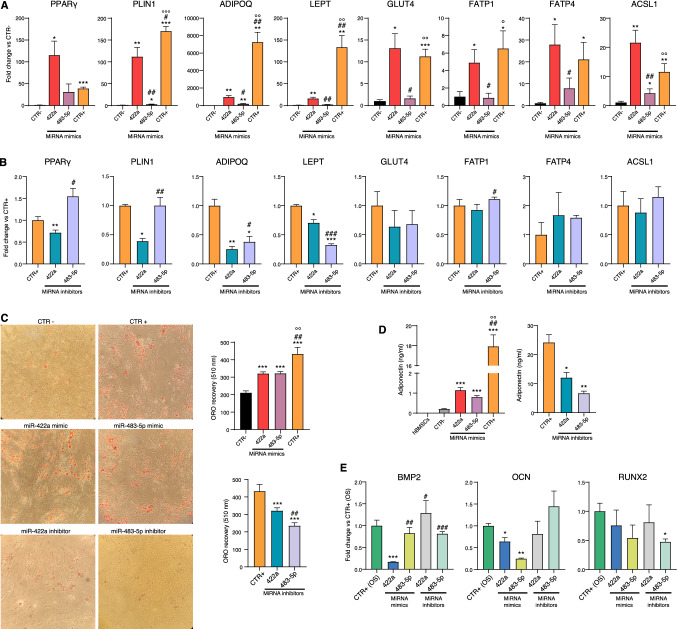
miR-422a and miR-483-5p promote adipogenesis in hBMSCs. **A** Adipogenesis-related mRNA fold change in hBMSCs transfected with miRNA mimics and negative miRNA mimic controls (CTR− and CTR +). CTR- indicates hBMSCs induced to differentiation in absence of indomethacin, while CTR + with indomethacin. MiRNA mimics were added to the adipogenic medium without indomethacin. * vs CTR−; # vs miR-422a mimic, ° vs miR-483-5p mimic. **B** Adipogenesis-related mRNA fold change in hBMSCs transfected with miRNA inhibitors. MiRNA inhibitors were added to the complete adipogenic medium. CTR + indicates hBMSCs treated with miRNA inhibitor negative control #1 and induced to adipogenesis with a complete adipogenic medium. * vs CTR + ; # vs miR-422a inhibitor. **C** Representative images and densitometric quantification of cells staining with Oil Red O. For mimic experiments: * vs CTR−; # vs miR-422a mimic, ° vs miR-483-5p mimic. For inhibitor experiments: * vs CTR + ; # vs miR-422a inhibitor. **D** Graph chart represents adiponectin released into the culture medium expressed in ng/ml. For mimic experiments: * vs CTR−; # vs miR-422a mimic, ° vs miR-483-5p mimic. For inhibitor experiments: * vs CTR + (**E**) Osteogenesis-related mRNA fold change in hBMSCs transfected with miRNA mimics and inhibitors of miR-422a and miR-483-5p. * vs CTR + ; # vs miR-422a mimic. Data are mean ± SD of three independent experiments. *, #, ° *t*-test *p* < 0.05; **, ##, °° *t*-test *p* < 0.01; ***, °°°*t*-test *p* < 0.001

To investigate the role of miR-422a in adipogenesis, we analyzed the expression of several specific molecular markers of adipogenesis in hBMSCs induced to differentiate for 14 days in the adipogenic medium in which miR-422a mimic was added instead of indomethacin, i.e. the component of the adipogenic cocktail exerting the greatest impact on the expression of key regulator genes of adipogenesis and lipid accumulation [35]. We also tested the effect of miR-483-5p, which we observed to be significantly up-regulated both in adipogenesis and osteogenesis (Fig. 3B) and whose role in promoting adipogenesis has already been demonstrated by previous reports [36]. We found that supplementation with miR-422a mimic induced a significant expression of the transcripts of all the genes tested (PPARγ, GLUT4, FATP1, FATP4, ACSL1, LEP, ADIPOQ, and PLIN1), in some cases (PPARγ, GLUT4, FATP1, FATP4, ACSL1) with an efficiency comparable to indomethacin (CTR +) (Fig. 4A). MiR-483-5p mimic promoted the upregulation of ACSL1, PLIN1, and ADIPOQ but less efficiently than miR-422a. qPCR analysis of intracellular miR-422a and -483-5p confirmed the efficiency of the transfection (Supplementary Fig. 3).

To strengthen these data, we further analyzed the effect of miR-422a or miR-483-5p inhibition during hBMSC differentiation by adding their specific antagomiRs to the complete adipogenic medium Notably, after 14 days of culture, the expression of PPARγ, PLIN1, LEP, and ADIPOQ was significantly reduced in miR422a-antagomiR transfected cells compared to adipogenic medium alone (CTR +). MiR-483-5p inhibition yielded similar results only for LEP and ADIPOQ (Fig. 4B).

The effect of both miR-422a and miR-483-5p on adipogenesis was further investigated by Oil Red O staining of hBMSC transfected with miRNA mimics or inhibitors in the same conditions described earlier (Fig. 4C). Lipid content is increased by miR-422a as well as by miR-483-5p mimic transfection compared to the adipogenic medium without indomethacin (CTR-). Accordingly, miR-422a or miR-483-5p inhibition by antagomirs significantly reduced lipid droplet formation induced by complete adipogenic medium (CTR +).

Since adiponectin is an essential factor secreted by mature adipocytes and miR-422a and miR-483-5p mimics alone were able to induce adiponectin (ADIPOQ) mRNA expression in hBMSC cultured in adipogenic medium without indomethacin (Fig. 4A), we assessed the amount of secreted adiponectin in this conditioned medium and compared its concentration with that released in the medium enriched with indomethacin (CTR +) or without miRNA mimics and indomethacin (CTR−). Both mimics of miR-422a and miR-483-5p significantly increased adiponectin release compared to negative control. Accordingly, miRNA inhibitors were able to reduce the amount of secreted adiponectin in the complete adipogenic medium (Fig. 4D).

As previously shown in Fig. 3, miR-422a is not modulated during hBMSC osteogenesis, contrary to miR-483-5p which is strongly up-regulated. Accordingly, forced expression of miR-422a inhibitor did not affect the expression of RUNX2, the master transcription factor for osteogenesis [37], and two other osteogenic-related genes—bone morphogenetic protein 2 (BMP-2) and osteocalcin (OCN)—after 14 days of pro-osteogenic conditions. However, miR-422a mimic caused a significant strong reduction of both BMP-2 and OCN gene expression (Fig. 4E). Interestingly, miR-483-5p inhibition reduced RUNX2 expression.

### MiR-422a and -483-5p are released from differentiating adipocytes and are present in plasma from subjects with osteoporosis

**Fig. 5:**
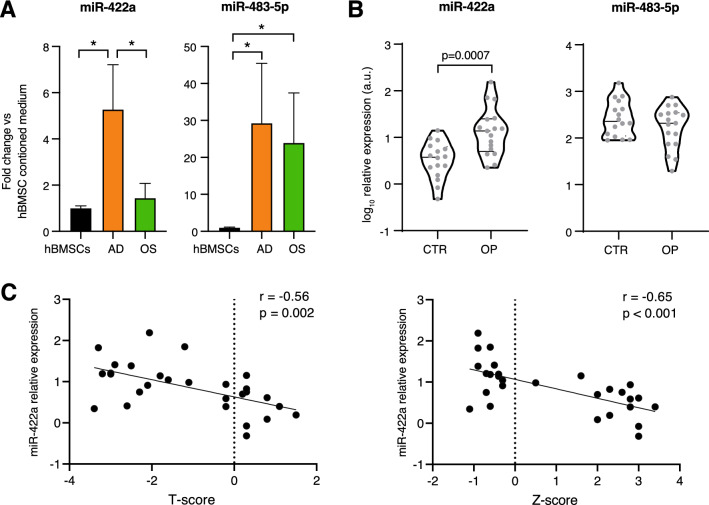
miR-422a has a higher expression in plasma of osteoporotic subjects compared with non-osteoporotic samples. **A** miRNA fold change in the culture medium of cells induced to differentiate into adipocytes (AD) and osteoblasts (OS) compared to hBMSCs. Data are mean ± SD of three independent experiments. *t-test *p* < 0.05. **B** Violin plots showing miRNA relative expression in plasma of non-osteoporotic (CTR) and osteoporotic (OP) subjects. **C** Scatter plot showing correlations between relative miR-422a expression levels (in arbitrary units, a.u.) and *T*-score or *Z*-score. Data are expressed as a mean of 2^−ΔCt^ normalized with *cel*-miR-39

To assess whether miRNAs related to hBMSC adipogenesis can be released in extracellular fluids we evaluated the levels of miR-422a and -483-5p in conditioned media harvested in the last 3 days out of the 14 days of culture in adipogenic and osteogenic medium. We observed that miR-422a level was significantly higher in the AD-conditioned medium compared to both undifferentiated hBMSC and OS-conditioned media, while miR-483-5p was similarly upregulated in both AD and OS-conditioned media compared to hBMSCs one (Fig. 5A).

To investigate the value of miR-422a and -483-5p as promising non-invasive candidate biomarkers in osteoporosis, we checked their circulating levels in subjects with primary type II osteoporosis (OP) and age-/gender-matched control subjects (CTR). The clinical and biochemical characteristics of the subjects are listed in Table 1. The levels of circulating miR-422a were higher in OP subjects compared to CTR (*p* = 0.0007), whereas no significant difference was shown for miR-483-5p (Fig. 5B). Finally, we analyzed the correlation between circulating miR-422a levels and bone mineral density assessed through dual-energy X-ray absorptiometry (DXA) in the whole cohort. Interestingly, we found significant negative correlations between circulating miR-422a and both T-score (*p* = 0.002) and *Z*-score (*p* < 0.001) (Fig. 5C).

**Table 1:** Biochemical and anthropometric characteristics of subjects enrolled for the study

Variables	Control subjects (*n* = 16)	Primary type II osteoporosis (*n* = 17)	*p*-Value
Age (years)	83.3 (5.9)	86.3 (3.5)	0.083
Gender (males, %)	7 (44%)	5 (29%)	0.392
BMI (kg/m^2^)	24.8 (2.0)	23.4 (2.5)	0.087
Creatinine (mg/dL)	1.3 (0.5)	1.4 (0.7)	0.642
Sodium (meq/L)	141.6 (3.5)	141.2 (4.9)	0.790
Potassium (meq/L)	4 (0.4)	3.9 (0.3)	0.421
Calcium (mg/dL)	8.9 (0.5)	8.6 (0.7)	0.169
Hemoglobin (g/dL)	12.4 (1.7)	11.0 (1.3)	0.012
ESR (mm/h)	39.6 (21.1)	52.4 (31.8)	0.186
CRP (mg/L)	2.9 (2.7)	3.8 (2.7)	0.346
*T*-score	0.3 (0.7)	−2.3 (1)	< 0.001
*Z*-score	2.4 (0.8)	−0.4 (0.8)	< 0.001
BMD	256.3 (485.6)	65.1 (249)	0.161

Data are presented as mean (SD)

*BMD* bone mineral density; *CRP* c-reactive protein; *ESR* erythrocyte sedimentation rate

*p* Values for unpaired *t* test (continuous variables) or Chi-squared test (categorical variables)

## Discussion

Adipogenesis is intimately linked to osteogenesis in the bone marrow milieu. Since bone and adipose tissue share a common origin, the identification of factors driving the hBMSC adipogenic program is of high relevance to human diseases characterized by disruption to the differentiation balance, such as osteoporosis and aging [38]. Interestingly, it has been suggested that MeCP2 plays a role in regulating both subcutaneous adipogenic process and, furthermore, osteogenesis in a rodent model of Rett syndrome [10, 39, 40], therefore, we hypothesized that it may represent a key factor addressing hBMSCs to one or the other differentiation pathway within the bone marrow.

In this work, we show that MeCP2 expression is modulated in an opposite way in adipocytes and osteoblasts both in vitro and in vivo and that its partial silencing in hBMSCs results in the induction of a pro-adipogenic transcriptional program. We demonstrate that MeCP2 modulation does not depend on different expression levels of its mRNA and accordingly on the methylation status of its gene, but on the expression of specific miRNAs. In fact, using a microarray approach, we have identified several differentially expressed miRNAs in hBMSC-derived adipocytes compared to undifferentiated cells. We focused on the most upregulated miRNAs, which could act as post-transcriptional repressors of MeCP2 expression during adipogenesis. Notably, miRNA-mRNA target prediction analysis aimed at identifying which of the most upregulated miRNAs could regulate MeCP2 expression showed that both miR-422a and miR-483-5p are able to target MeCP2 mRNA. However, since miR-422a is the only one modulated in adipogenesis but not in osteogenesis, we thought it may be the right candidate to reduce MeCP2 expression in adipocytes. We found for the first time that forced expression of miR-422a and miR-483-5p in hBMSC can significantly downregulate MeCP2 expression and promote adipogenesis, with miR-422a being more efficient in inducing the expression of adipogenic markers, including adiponectin. Albeit the concentration of adiponectin released by cells treated with miRNA mimics did not reach the levels of positive control (adipogenic medium with indomethacin), we observed a significant > fivefold increase compared to cells treated with the medium without indomethacin. Indeed, indomethacin is the major driver of adiponectin synthesis in in vitro models of adipogenesis [41]. Interestingly, data obtained with miR-483-5p are in agreement with those found by others on its ability to inhibit MeCP2 expression during fetal development [32] and to regulate adipogenesis in the subcutaneous environment [36]. On the contrary, miR-422a and miR-483-5p inhibition rescues MeCP2 expression in adipocytes and reduces adipogenic marker expression. Interestingly, an in vitro study on human visceral preadipocytes showed that metformin inhibits adipogenesis with a concomitant reduction of miR-422a levels [42]. More in general, miR-422a increased during adipogenesis, exerted a greater impact on the adipogenic process compared to miR-483-5p and induced a significant inhibition of the osteogenic markers BMP-2 and osteocalcin. Overall, these observations suggest that miR-422a is specifically boosted in adipogenesis and may possibly inhibit osteogenesis, a hypothesis that deserves future investigations.

The great effect of miR-422a and miR-483-5p on adipogenesis may be related at least in part to the downregulation of MeCP2 expression in differentiating hBMSCs. Indeed, partial silencing of MeCP2 in undifferentiated mesenchymal cells led to the acquisition of an adipogenic profile with upregulation of PPARγ and PLIN1 mRNAs, independently from miR-422a and miR-483-5p. Of note, the expression of miR-422a and miR-483-5p does not depend on MeCP2 itself because they are not modulated by its silencing. Furthermore, this effect is also confirmed by the upregulation of adiponectin, leptin, FABP4, and PLIN1 mRNAs in silenced cells harvested in an adipogenic medium compared to cells transfected with an empty lentiviral vector. However, in this condition, we did not observe an effect on the mRNA levels of the transcription factor PPARγ, which may be covered by the strong induction of the adipogenic cocktail. In agreement with our findings, RTT patients show high levels of circulating leptin [43, 44] and adiponectin [45]. Adiponectin, one of the most extensively studied adipocyte-derived factors, exerts a plethora of beneficial effects through its insulin-sensitizing, anti-atherogenic, and anti-cancer properties [46, 47]. Since MAT appears to be the major contributor to circulating adiponectin, it has been suggested that its increase may have beneficial effects in compromised health conditions such as anorexia nervosa and chemotherapy although this increase is related to a condition of osteoporosis [14].

In fact, one of the proposed mechanisms in the pathogenesis of osteoporosis is a shift of hBMSC differentiation toward adipocyte rather than osteoblast [48]. For this reason, it is important to characterize the broad range of mediators in the bone marrow milieu that can regulate the commitment of MSCs. Studies on RTT patients and different RTT mouse models highlighted the existence of a direct relationship between MeCP2 loss of function and the alteration of bone homeostasis, which contributes to the onset of osteoporosis and to a higher risk of bone fracture [10, 49–51]. Importantly, a mouse model in which MeCP2 has been reactivated specifically in the nervous system but remained silenced elsewhere showed that bone abnormalities are due to a loss of MeCP2 in peripheral tissues [52], confirming a metabolic component in RTT syndrome. Interestingly, a recent study showed that the overexpression of MeCP2 in BMSCs enhanced the expression of osteogenic markers, including RUNX2 and osteocalcin, and promoted calcium deposition in a mouse model of osteoporosis [53]. Here, we showed that not only MeCP2 genetic silencing can affect bone biology but even that specific miRNAs affecting MeCP2 expression, i.e. miR-422a and -483-5p are capable to influence osteogenesis when hBMSCs were cultured under proper conditions. Besides MeCP2, miR-422a significantly reduced mRNA levels of other specific targets involved in bone biology, BMP2, and osteocalcin, while miR-483-5p inhibitor induced a decline in RUNX2 mRNA levels. Accordingly, a recent report showed that intra-articular injection of miR-483-5p inhibitor delays the development of osteoarthritis through the reduction in the number of RUNX2-positive chondrocytes [54]. Nonetheless, miR-483-5p resides in an intron of the IGF2 gene and it has been shown to upregulate the expression of its host gene [55], which is involved in longitudinal and appositional bone growth [56]. Overall, these data suggest that both miRNAs are somehow involved in the commitment of hBMSCs, with miR-422a exerting a more pronounced effect toward the adipogenic lineage. In addition, miR-422a showed a higher expression in the plasma of osteoporotic patients compared with non-osteoporotic controls and was negatively related to *T*-score and *Z*-score. A previous study showed that the search for circulating miRNAs as minimally invasive biomarkers for osteoporosis revealed that miR-422a is upregulated in circulating monocytes from low BMD postmenopausal women [57]. De-Ugarte and colleagues showed significant overexpression of miR-483-5p through a microRNA array on bone samples from postmenopausal women with a history of osteoporotic fractures [58]. However, this latter observation was not replicated in our cohort, probably due to the limited number of patients and to the different forms of osteoporosis (type II, age-related vs. type I, postmenopausal) considered.

In conclusion, we showed that miR-422a and miR-483-5p act as pro-differentiation factors in hBMSCs and that these two miRNAs can affect the adipogenesis process by influencing MeCP2 expression. Our findings emphasize the need to unravel MeCP2 expression and regulation in peripheral tissues, especially in bone marrow stromal cells, with a look at the potentially related diseases. A thorough comprehension of the factors capable to affect hBMSC differentiation is important not only in the context of bone mineral disease. Many efforts have been devoted to preventing metabolic complications due to the accumulation and increased secretory activity of visceral adipose tissue. Of note, BM adipose tissue sustains its own integrity through the release of extracellular vesicles (EVs) containing a typical adipocyte signature, as well as anti-osteoblastic miRNAs exerting their effects on the nearby hBMSCs [59]. It is tempting to speculate that such EVs could be secreted also in the bloodstream, affecting adipose tissue homeostasis at the systemic level. In this regard, the study of EVs as mediators of the extracellular dynamics of miR-422a and miR483-5p represents an intriguing future perspective for the present research. Future investigations are warranted to disentangle the roles of miRNAs showing opposite patterns of modulation during adipogenesis and to understand how these miRNAs integrate into a complex network regulating adipose tissue formation and function.

## Materials and methods

### Cell culture and differentiation

Human bone marrow stromal cells (hBMSCs) were purchased from Lonza (Allendale, NJ, USA) and maintained in α-MEM (Euroclone, 20016, Milano, Italy) supplemented with 10% fetal bovine serum (FBS) (Lonza), 2 mM l-glutamine, 100 U/ml penicillin, 100 mg/ml streptomycin. Experiments were performed using different batches of BMSCs up to the fifth passage.

For adipogenic differentiation, BMSCs were cultured as shown in Rippo et al. [60]. Briefly, hBMSCs were seeded at 8 × 10^3^ cells/cm^2^ on six-well plates in AD containing complete a-MEM, supplemented with 0.5 mM dexamethasone, 5 mg/ml insulin, 0.45 mM isobutylmethylxanthine (IBMX), and 0.2 mM indomethacin (Sigma-Aldrich, St. Louis, MO, USA). In specific miRNA mimics experiments, the adipogenic medium was supplemented with miR-422a and miR-483-5p mimics instead of 0.2 mM indomethacin. In these sets of experiments, we used two different adipogenic cocktails as controls, with (CTR +) or without indomethacin (CTR−).

Dedifferentiation of adipocytes obtained from three different subcutaneous fat tissue human samples was obtained as previously described in Poloni et al. [33]. BM-derived mesenchymal cells and adipocytes were collected from patients undergoing hip surgery and maintained in their growth medium until analysis.

All tissue samples were collected in accordance with local ethics committee guidelines (300/DG), and all participants provided their written informed consent to take part in the study. During the dedifferentiation process, mature adipocytes lost their lineage gene expression profile, assumed the typical mesenchymal morphology and immunophenotype, and expressed stem cell genes.

### Adipocyte staining

Adipocyte differentiation was assessed by Oil Red O (ORO) staining. Briefly, cells were washed with PBS and fixed with 4% paraformaldehyde for 5 min. After fixation, samples were washed twice in PBS, followed by incubation with freshly filtered ORO staining solution (six parts Oil Red O stock solution and four parts H2O; Oil Red O stock solution is 0.5% Oil Red O in isopropanol) for 30 min. For quantitative analysis, ORO was extracted from the cells with isopropanol and quantified spectrophotometrically at 520 nm.

### RNA extraction and RNA expression

Total RNA was recovered from hBMSCs using the Total RNA Purification Kit purchased from Norgen (#37500, Norgen Biotek, Thorold, ON, Canada) which allows the isolation of both microRNAs and mRNAs. RNA was used immediately or stored at −80 °C until analysis by Real-Time (RT)-PCR.

#### mRNA analysis

1 µg of RNA was transcribed into cDNA using PrimeScript™ RT Reagent Kit with gDNA Eraser (RR047A, Takara Bio) according to the manufacturer’s instructions. One-twentieth of first-strand cDNA was used as a template for RT-PCR amplification. RT-PCR was performed with TB Green Premix Ex Taq (Tli RNase H Plus) (RR420A, Takara Bio) in a reaction volume of 10 µl with specific primers according to the protocol. β-actin or/and IPO8 was used as reference gene. Primers were listed in Supplementary Table 1.

#### MicroRNA analysis

MiRNAs were reverse transcribed following the manufacturer’s instructions (#4366596, Thermo Fisher Scientific) using specific stem-loop primers for each miRNA. The RT-PCR reaction mix included TaqMan MicroRNA assay (#4427975, Thermo Fisher Scientific), TaqMan Universal Master mix no UNG (4440040, Thermo Fisher Scientific) and RT product. RNU48 was used as a reference gene. The 2^−ΔCT^ method was used to determine miRNA expression.

### TaqMan MicroRNA array analysis of mature microRNAs

Microarray analysis was performed as previously described [61, 62]. In brief, the previously isolated RNA was reverse transcribed by priming with a mixture of looped primers using the manufacturer’s instructions (Megaplex RT primers Human Pool A v2.1, Thermo Fisher Scientific). Pre-amplification of cDNA was performed using TaqMan Preamp Master Mix (#4384266, Thermo Fisher Scientific) and Megaplex PreAmp Primers (10×), Human Pool A v2.1 (#4399233, Thermo Fisher Scientific). Pre-amplified cDNA was used for mature miRNA profiling by RT-PCR instrument equipped with a 384-well reaction plate (7900 HT, Applied Biosystems) and TaqMan Array Human MicroRNA Cards v2.0 pool A (#4398977, Thermo Fisher Scientific) containing 367 different human miRNA assays in addition to selected small nucleolar RNAs. miRNAs expressed (Ct ≤ 30) in at least one condition (hBMSCs, adipogenesis) were included in the analysis. Data were presented as log_2_ fold change versus undifferentiated hBMSCs.

### miRNA target prediction analysis

The open-source encyclopedia of RNA interactomes (http://starbase.sysu.edu.cn/index.php) [63] was utilized to locate target genes of miR-422a and miR-483-5p.

### Cell transfection with miRNA mimics and inhibitors

Transfection of miRNA inhibitors and mimics was conducted as previously described [64]. Briefly, 1 × 10^5^ hBMSCs were plated in six-well plates and incubated overnight before transfection with miR-422a and miR-483-5p miRVANA miRNA mimics (MC12541, MC12629), miRVANA miRNA inhibitors (MH12541, MH12629), miRVANA miRNA inhibitor negative control #1 (4464077) or with miRVANA miRNA mimic negative control #1 (4464058, all from Thermo Fisher Scientific, San Jose, CA, USA) at a concentration of 30 nM. Transient transfection was performed using TransIT-2020 transfection reagent (MIR 5404, Mirus Bio LLC, Madison, WI, USA), according to the manufacturer’s protocol. The ratio of transfection reagent (µl)/miR (µg) equal to 2:1 was found to be optimal. Transfection was carried out concomitantly with the induction of differentiation and repeated at every medium replacement. Analyses on adipogenesis induction were performed after 14 days after transfection.

### Luciferase reporter assay

The luciferase reporter assay was performed by Creative Biogene Biotechnology (Shirley, NY, USA). The wild-type MeCP2 reporter (MeCP2-WT) and the mutant MeCP2 reporter (MeCP2-Mut) were generated by subcloning the 3′-UTR sequences of MeCP2 bracketing the predicted miR-422a-5p binding site and the full-length sequences of MeCP2-Mut into the *Xho*I/*Xba*I site located at 3′UTR of pmirGLO (Promega) vectors. The MeCP2 3′-UTR sequences were as follows: WT human MECP2 3′UTR sequence (miRNA binding sites in italics), CGACCTTGACCTCACTCAGA*AGTCCAGA*GTCTAGCGTAGTGCAGCAGGGCAGTAGCGGTAATACTTAGTCAAATGTAATGTGGCTTCTGGAATCATT*GTCCAGA*GCTGCTTCCCCGTCAC; mutant human MECP2 3′UTR sequence (mutant sites in italics), CGACCTTGACCTCACTCAGA*TCAGGTCT*CAGATCCGTAGTGCAGCAGGGCAGTAGCGGTAATACTTAGTCAAATGTAATGTGGCTTCTGGAATCATT*CAGGTCT*GCTGCTTCCCCGTCAC. After ligation of the WT/mutant human MECP2 3′UTR fragments into linearized pmirGLO by recombination reaction, HEK293T cells were transfected for 48 h with the pmirGLO-UTR reporter plasmid in combination with Negative Control (NC, sequence UUCUCCGAACGUGUCACGUUU) mimics or miR-422a-5p mimics (sequence: ACUGGACUUAGGGUCAGAAGGC) at a final concentration of 20 nM in 25 μl of pure DMEM. The Firefly luciferase activity, normalized to Renilla luciferase (for transfection efficiency), was determined with the dual-luciferase reporter assay system (Promega), according to the manufacturer’s instructions, and reported as % of the negative control mimic activity.

### Adiponectin production

Cell medium was collected at the end of the experiments, centrifugated, and stored at −80 °C until used in the assay. Adiponectin concentration was measured using a commercially available high-sensitivity enzyme-linked immunosorbent assay (ELISA) (AG-45A-0001YEK-KI01, AdipoGen).

### Von Kossa staining

After osteogenic induction for 21 days, cells were fixed in 4% paraformaldehyde for 20 min before being stained with 5% aqueous silver nitrate solution for 45 min at room temperature under the light. Next, the samples were washed with deionized water and stained with 5% sodium thiosulfate for 10 min.

### Specimen collection and immunohistochemistry

Small fragments of human femoral bone were collected from patients undergoing hip surgery and then fixed in buffered formalin 10% for 24–48 h. All tissue samples were collected in accordance with local ethics committee guidelines 300/DG, and all participants provided their written informed consent to take part in the study.

After a decalcification step in neutral EDTA-sodium hydroxide, a conventional paraffin embedding procedure was performed. Inguinal and omental adipose tissue and femoral BM aspirates were obtained from adult female Sprague–Dawley albino rats (*n* = 3, 190–220 g; age, 3 months; Charles River, Milan, Italy). Experiments were carried out in accordance with the Council Directive 2010/63EU of the European Parliament and the Council of September 22, 2010, on the protection of animals used for scientific purposes and approved by the local veterinary service. Samples were fixed in 4% paraformaldehyde overnight at 4 °C, then paraffin-embedded. Subsequently, 3 µm sections were obtained from all the specimens and used for the detection of MeCP2 reactivity. Briefly, following antigen retrieval, tissues were blocked in 3% H_2_O_2_ for 15 min at room temperature, washed, and then probed with rabbit polyclonal anti-MeCP2 antibody (abcam #2828. Cambridge, UK) 1:200 overnight at 4 °C in a humidified chamber. Tissues were washed extensively in PBS and detection was performed using an HRP-conjugated secondary antibody followed by DAB colorimetric detection using a kit (Cell Signalling Technology. MA, USA). Tissues were counterstained with hematoxylin, dehydrated, and mounted. Images were taken using a Nikon Eclipse 80i microscope.

### Methylation analysis

Genomic DNA was extracted from hBMSCs and hBMSC-derived adipocytes and osteoblasts using Qiagen’s QiAmp mini kit following the manufacturer’s recommendations. Each sample type was extracted and assessed for its DNA methylation profile in triplicate. Briefly, 1 µg of DNA was converted with bisulfite using the EZ DNA Methylation Kit (#D5001, Zymo Research) and analyzed using the Infinium HumanMethylationEPIC BeadChip (#20042130, Illumina), which allows assessing the methylation status of more than 850,000 CpG sites across the genome. Raw data files were extracted using the *minfi* Bioconductor package (CIT Preprocessing, normalization, and integration of the Illumina HumanMethylationEPIC array with minfi). Quality check resulted in the removal of 1536 probes having a detection *p*-value < 0.05 in more than 1% of the samples and in the removal of 98,855 potentially cross-reactive probes according to Zhou et al. (CIT Comprehensive characterization, annotation and innovative use of Infinium DNA methylation BeadChip probes). Normalization was performed using the *preprocessFunnorm* function implemented in *minfi* and DNA methylation was expressed as beta values ranging from 0 (0% of methylation) to 1 (100% of methylation). DNA methylation values in hBMSCs and hBMSC-derived adipocytes and osteoblasts were compared pairwise using the *limma* package and *p*-values were adjusted using the Benjamini–Hochberg procedure. Adjusted *p*-values < 0.01 were retained as significant. For the analysis of MeCP2 DNA methylation, the genomic region encompassing the gene plus 5000 bp upstream and downstream (chrX:153282264–153368188, hg19 assembly) was considered.

### Lentivirus construction and infection

LentiLox 3.7 (pLL3.7) vector system was used to induce RNA interference of MeCP2. Three different short hairpin RNA (shRNA) sequences targeting MeCP2 transcript (MeCP2sh1, MeCP2sh2, and MeCP2sh3—see Supplementary Table 2 for target sequences) were cloned into pLL3.7 as described [65]. Lentiviruses were produced by co-transfecting 293T cells with shRNA-containing pLL3.7 plasmids, or pLL3.7 empty vector, in combination with packaging plasmids as described [66]. Supernatants of transiently transfected 293T cells were recovered after 36 h and two cycles (6 h each) of infection of hBMSCs were performed within 48 h with a pool of the three shRNA-containing lentiviral vectors. EGFP positivity of target cells was monitored to verify the efficiency of infection which approximately reached 90%.

### Protein extraction and immunoblotting

Total protein was extracted using RIPA buffer (150 mM NaCl, 10 mM Tris, pH 7.2, 0.1% SDS, 1.0% Triton X-100, 5 mM EDTA, pH 8.0) containing protease inhibitor cocktail (Roche Applied Science, Indianapolis, IN, USA) and quantified using the Bradford method. Proteins were separated on gradient SDS-PAGE gels and transferred to nitrocellulose membranes (Whatman). Membranes were then incubated with the primary antibodies overnight at 4 °C. The following primary antibodies were used: MeCP2 (D4F3) XP Rabbit (#3456, Cell Signaling Technology); β-Actin (8H10D10) Mouse mAb (#3700, Cell Signaling Technology); PPARγ (81B8) Rabbit (#2443, Cell Signaling Technology). After incubation with the specific HRP-conjugated antibody (Vector; 1:10,000 dilution), the chemiluminescent signal was detected using Clarity and/or Clarity Max (Bio-Rad, Italy) and images were acquired with Alliance Mini HD9 (Uvitec, Cambridge, UK). Densitometric analysis was performed with ImageJ software (https://imagej.nih.gov/ij/download.html). Full and uncropped Western Blots were provided as Supplemental Material.

### Plasma samples

Plasma samples were obtained from 16 healthy subjects (CTR) and 17 osteoporotic patients (OS) enrolled in the SAFARI study. Subjects were considered healthy if they did not present osteoporosis, liver diseases, renal failure, history of cancer, neurodegenerative disorders, infectious or autoimmune diseases. Samples were collected at the Ospedali Riuniti Marche Nord (Fano, Italy) hospital facilities. The procedure was approved by the Ethical Committee Regione Marche (CERM). Written informed consent was collected from all participants.

### Statistical analysis

Data are presented as mean ± standard deviation (SD) of at least three independent experiments. The Student’s *t*-test was applied to determine differences between samples. The correlation between circulating miR-422a levels and the *Z*-score and *T*-score score was assessed using Pearson’s correlation coefficient. Probability (*p*) values lower than 0.05 were considered statistically significant. The reported *p*-values were two-tailed in all calculations. Data were analyzed with SPSS 25.0 (SPSS Inc., IBM, Chicago, IL, USA).

### Supplementary Information

Below is the link to the electronic supplementary material.

Supplementary file1 (PDF 1821 KB)

## Data Availability

Complete profiling results of hBMSCs miRNAs have been deposited in NCBI’s Gene Expression Omnibus (GEO) (https://www.ncbi.nlm.nih.gov/geo) with accession reference GSE189508.
