# Supplementary material for: MiR-422a promotes adipogenesis via MeCP2 downregulation in human bone marrow mesenchymal stem cells
Source: Cell Mol Life Sci. 2023 Feb 27;80(3):75. doi: 10.1007/s00018-023-04719-6 (PMC9971129; doi:10.1007/s00018-023-04719-6)
Supplement: Supplementary file 1 — Supplementary file1 (PDF 1821 KB) [file 18_2023_4719_MOESM1_ESM.pdf]

MiR-422a promotes adipogenesis via MeCP2 downregulation in human bone marrow mesenchymal stem cells

SUPPLEMENTARY INFORMATION

Supplementary Figure 1. Characterization of human bone marrow stromal cell (A) adipogenic and (B) osteogenic differentiation markers.

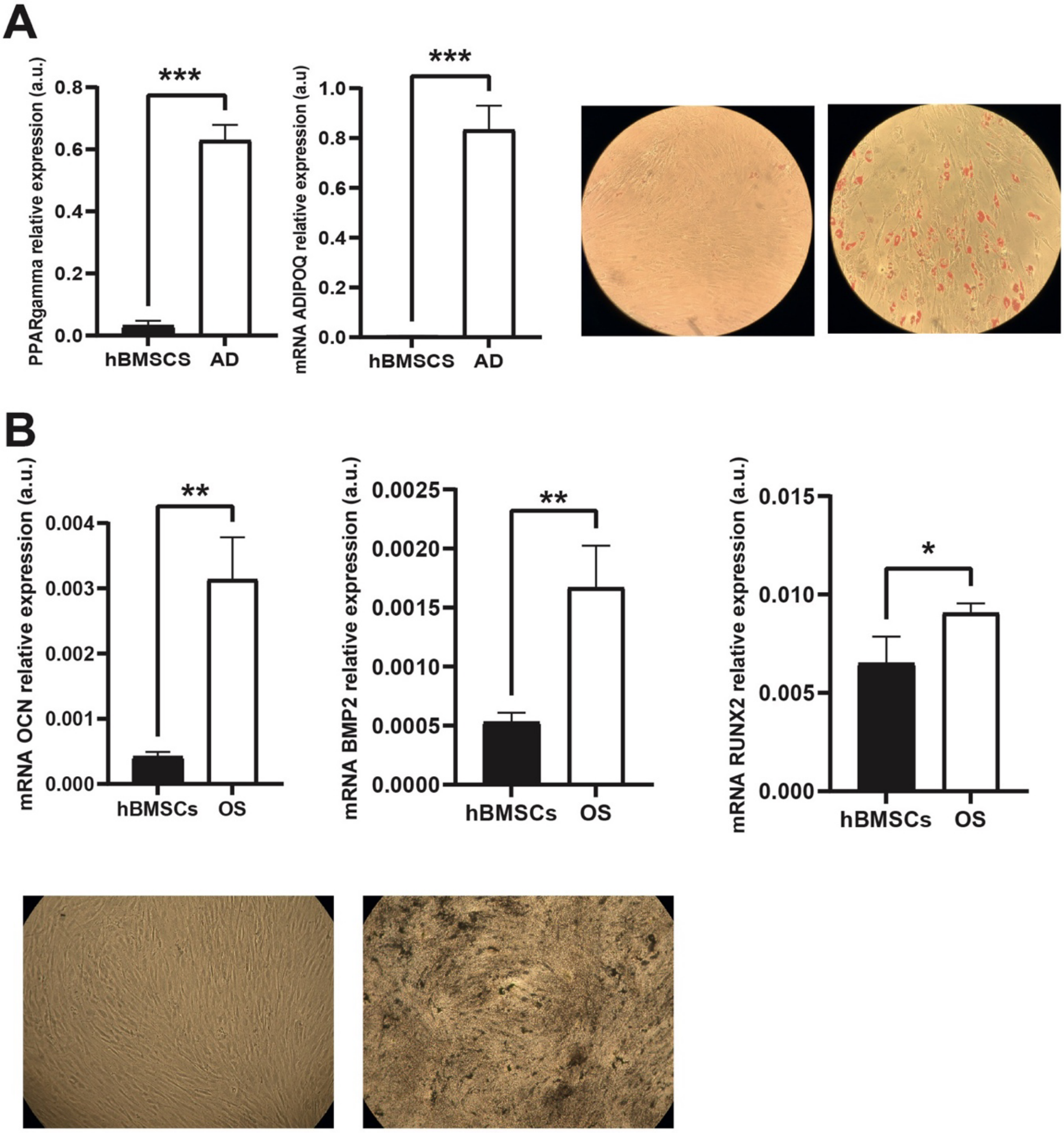

Supplementary Figure 2. MiR-422a and miR-483-5p relative expression (expressed in a.u.) in hBMSCs infected with shRNA-containing (sh-MeCP2) or empty (EV) lentiviral vectors. Data were normalized to RNU48 expression.

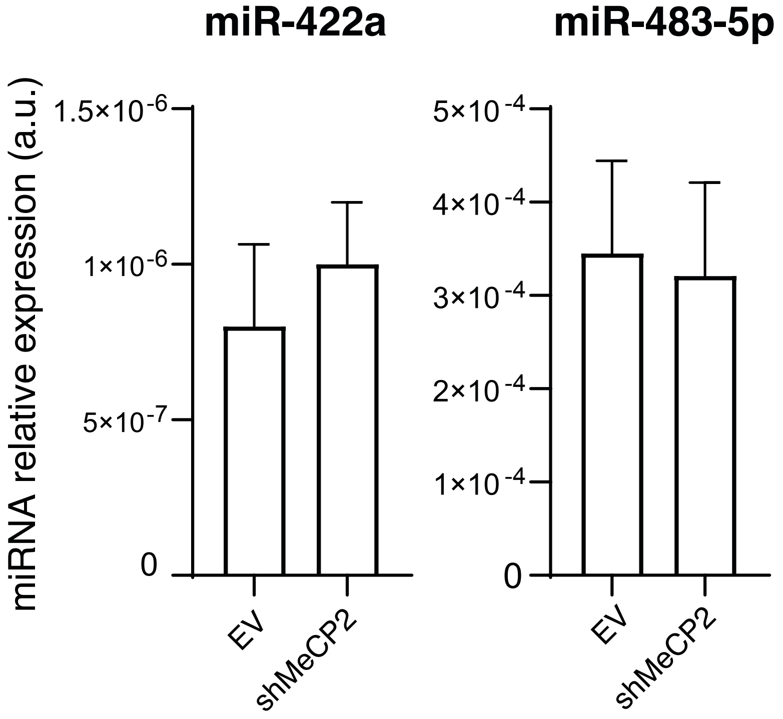

Supplementary Figure 3. Assessment of miR-422a and miR-483-5p mimic transfection efficiency through qRT-PCR in hBMSCs (expressed in a.u.).

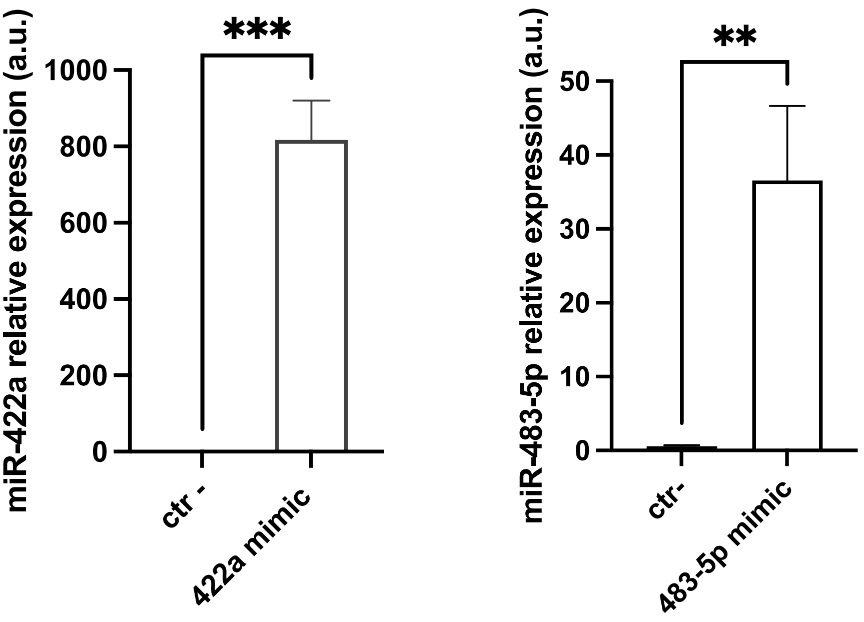

Supplementary Table 1.

Supplementary Table 1 provides a list of the primer sequences used.

	**Forward primer (5'-3')**	**Reverse primer (5'-3')**
IPO8	CGTTCCTCCTGAGACTCTGC	GAATGCCCACTGCATAGGTT
BETA-ACTIN	AAACTGGAACGGTGAAGGTG	CAAGGGACTTCCTGTAACAATGC
PPARGAMMA	AGCCTCATGAAGAGCCTTCCA	ACCCTTGCATCCTTCACAAGC
FATP1	GGTGGTTCAGTACATCGGGG	GAACTCCTCCCAGATGGCAG
FATP4	CCCAGGTGGCTGAGTTCTAC	GGATGGGGTACACGAAGGAC
Adiponectin	CCTAAGGGAGACATCGGTGA	GTAAAGCGAATGGGCATGTT
Leptin	GGCTTTGGCCCTATCTTTTC	CCAAACCGGTGACTTTCTCT
Acsl-1	CATGAAGGCGATGGAGGACC	GGGGTTGCCTGTAGTTCCAC
Plin1	GAAAAGATTCCCCGCCCTCC	CTGATGCTGTTTCTGGCACTG
MeCP2	CACGGAAGCTTAAGCAAAGG	CATTAGGGTCCAGGGATGTG
Glut4	CGTCGGGCTTCCAACAGATA	CACCGCAGAGAACACAGCAA
FABP4	TCACCTGGAAGACAGCTCCT	AAGCCCACTCCCACTTCTTT
Bmp2	GGAATGACTGGATTGTGGCT	TGAGTTCTGTCGGGGACACAG
Ocn	ACCTGTATCAATGGCTGGGAG	TCAGCCAACTCGTCACAGTC
Runx2	AGATGGGACTGTGGTTACTG	GTAGCTACTTGGGGAGGATT

Supplementary Table 2.

Supplementary Table 2 provides a list of the sequences targeted by the different shRNAs on MeCP2 transcript.

shRNA name	MeCP2 target sequence
MeCP2sh1	5'-GGAAGCTCCTTGTCAAGAT-3'
MeCP2sh2	5'-TCAGGCCATTCCCAAGAAA-3'
MeCP2sh3	5'-AGACCGTACTCCCCATCAA-3'
